# Radiofrequency Exposure Amongst Employees of Mobile Network Operators and Broadcasters

**DOI:** 10.1093/rpd/ncw283

**Published:** 2016-10-13

**Authors:** Ian Litchfield, Martie van Tongeren, Tom Sorahan

**Affiliations:** 1 Institute of Applied Health Research, College of Medical and Dental Sciences, University of Birmingham, Edgbaston, Birmingham B15 2TT, UK; 2 Institute of Occupational Medicine, Research Avenue North, Riccarton, Edinburgh EH14 4 AP, UK

## Abstract

Little is known about personal exposure to radiofrequency (RF) fields amongst employees in the telecommunications industry responsible for installing and maintaining transmitters. IARC classified RF exposure as a possible carcinogen, although evidence from occupational studies was judged to be inadequate. Hence, there is a need for improved evidence of any potentially adverse health effects amongst the workforce occupationally exposed to RF radiation. In this study, results are presented from an exposure survey using data from personal monitors used by employees in the broadcasting and telecommunication industries of the UK. These data were supplemented by spot measurements using broadband survey metres and information on daily work activities provided by employee questionnaires. The sets of real-time personal data were categorised by four types of site determined by the highest powered antenna present (high, medium or low power and ground-level sites). For measurements gathered at each type of site, the root mean square and a series of box plots were produced. Results from the daily activities diaries suggested that riggers working for radio and television broadcasters were exposed to much longer periods as compared to colleagues working for mobile operators. Combining the results from the measurements and daily activity diaries clearly demonstrate that exposures were highest for riggers working for broadcasting sites. This study demonstrates that it is feasible to carry out exposure surveys within these populations that will provide reliable estimates of exposure that can be used for epidemiological studies of occupational groups exposed to RF fields.

## INTRODUCTION

The growing prevalence of mobile phones and their associated infrastructure has brought concerns about adverse health effects^1–6^ to the forefront of public and political concern^7–10^. Recently, IARC classified radiofrequency (RF) as a possible carcinogen based primarily on ‘limited’ evidence for an association of glioma and acoustic neuroma with mobile telephone use, although it described evidence in relation to the occupationally exposed as ‘inadequate’^11^. Large scale studies continue to explore the association between RF exposure and adverse health effects^12^. This is likely to generate renewed interest in the effects of RF on the human body. However the accurate monitoring of personal RF exposure remains a significant inhibitive factor, particularly in complex work environments that can contain a wide range of sources, powers and frequencies, a challenge that has been widely acknowledged^13^.

Some of the most powerful emitters of RF can be found in the telecommunications and broadcasting industries and therefore the exposure of the workers they employ is of particular interest. The nature of occupational exposure within these working environments is dependent upon the RF and power of the antennas they encounter, the physical characteristics of the environment where they are housed, and the regularity with which they are visited. As a result, and dependent upon their roles and responsibilities, employees can experience exposures of varying duration and intensity.

Only a limited amount of research has been published on occupational RF exposures of workers in the telecommunication and broadcast industries^14–16^. Though the combination continues to evolve, the types of RF source have been established for some time and here results are presented from a study that explored the exposure of telecommunication and broadcast employees in the UK in the early 2000 s^14^.

## METHODS

### Settings

This work involves those employed either by mobile network operators, or the broadcasting industry in the UK. The network operators provide the infrastructure to support services across the GSM networks using antennas located singly or with other antennas on a range of structures including towers, masts and roof-tops. These antennas can be sector or omnidirectional, operate at frequencies of 900 and 1800 MHz, and have individual powers of up to 10 W, which could add up to 70 W for heavily used sites. The broadcasters distribute audio and/or video signals via antennas that are commonly housed on purpose built structures. They use medium, high, very high-frequency (VHF) and ultra high-frequency (UHF) systems ranging from 3 MHz up to 3.0 GHz and radiated power up to 200 kW.

### Participants

Those employees whose exposure could potentially exceed International Commission on Non-ionising Radiation Protection (ICNIRP) reference levels for public exposure^17^ were invited to participate. Senior managers at each company helped identify the job titles of eligible staff. Those whose RF exposure was solely as a result of mobile phone usage or from using wireless local networks were excluded. Those eligible received an information leaflet containing information on the study and allowing them to provide informed consent.

### Data collection

Data that were collected by I.L. , and first analysed by Cooper *et al*.^14^ are presented. In collecting these data, two designs of personal monitors were used over the course of the study. The majority of measurements was made with the Wandel & Goltermann ESM-20, designed to measure field strengths and then present them as a percentage of the ICNIRP^17^ reference levels. They were sensitive to electric fields 1 MHz to 40 GHz^10^. During the latter stages of the study, the Nardalert XT was used, which was both sensitive to a broader range of frequencies, i.e. 100 kHz to 100 GHz, and could measure electric field strength levels down to 10% of ICNIRP guidelines^18^ (see Table 1)^17^. Each data point is the average of 20 measurements taken over a 2 s period. The monitors were worn on the breast pocket of each participant and a member of the study team was present each time a monitor was worn to ensure this location was consistent for all participants. To further characterise the exposure regimes experienced by cohort, a series of spot measurements in the vicinity of telecommunication antennas and UHF and VHF broadcast antennas were also conducted using three different types of broadband survey metre; the Holaday HI-4417, and the Narda 8716 and 8712 survey metres.

**Table 1.** ICNIRP guidelines for exposure to time varying electric and magnetic fields for frequencies up to 10 GHz.

Exposure characteristics	Frequency range	Current density for head and trunk (mA m22) (rms)	Whole-body average SAR (W kg21)	Localised SAR	
				(head and trunk) (W kg21)	(limbs) (W kg21)
Occupational exposure	Up to 1 Hz	40	—	—	—
	1–4 Hz	40/*f*	—	—	—
	4 Hz–1 kHz	10	—	—	—
	1–100 kHz	*f*/100	—	—	—
	100 kHz–10 MHz	*f*/100	0.4	10	20
	10 MHz–10 GHz	—	0.4	10	20

Notes: 1. *f* is the frequency in Hz. 2. Because of electrical inhomogeneity of the body, current densities should be averaged over a cross-section of 1 cm^2^ perpendicular to the current direction. 3. For frequencies up to 100 kHz, peak current density values can be obtained by multiplying the rms value by *u*2(1.414). For pulses of duration *t*_p_ the equivalent frequency to apply in the basic restrictions should be calculated as *f*_5_ 1/(2*t*_p_). 4. For frequencies up to 100 kHz and for pulsed magnetic fields, the maximum current density associated with the pulses can be calculated from the rise/fall times and the maximum rate of change of magnetic flux density. The induced current density can then be compared with the appropriate basic restriction. 5. All SAR values are to be averaged over any 6-min period. 6. Localised SAR averaging mass is any 10 g of contiguous tissue; the maximum SAR so obtained should be the value used for the estimation of exposure. 7. For pulses of duration *t*_p_ the equivalent frequency to apply in the basic restrictions should be calculated as *f*_5_ 1/(2*t*_p_). Additionally, for pulsed exposures in the frequency range 0.3–10 GHz and for localised exposure of the head, in order to limit or avoid auditory effects caused by thermoelastic expansion, an additional basic restriction is recommended. This is that the SA should not exceed 10 mJ kg21 for workers and 2 mJ kg21 for the general public, averaged over 10 g tissue.

To capture data on the typical length of exposure to RF for different groups of workers across both industries, questionnaires were distributed to employees in each company. These captured information on the typical activities during the working day including duration of working in areas with possible RF exposure. Each individual received 10 questionnaires to account for two working weeks.

### Analysis

The data recorded by personal monitors were collated by type of site visited and Uncensor 4.0^19^ software was used to produce measures of central tendency, and to carry out a root mean square (rms) analysis. Data on work activity from each company were collated and produced descriptive statistics of the amount of time spent working in proximity to RF emitters by sector and job type.

## RESULTS

### Telecommunication sites

A total of seven sites primarily used for telecommunications were visited and spot measurements undertaken. Additional radio systems were also located at some of these sites including omnidirectional paging antennas and point-to-point microwave links. The strongest ambient field strengths were found near pager antennas. The field strengths from dish antennas were commonly below the detection threshold of the instrumentation. The measurements are summarised in Table 2.

**Table 2.** Electric field strength measured in the vicinity of telecommunications antennas.

Location	Electric field strength (V m^–1^)						
	Site 1	Site 2	Site 3	Site 4	Site 5	Site 6	Site 7
Ambient level above platforms/roof top	1–20	8–50	10–40	≤17	3–5	4–12	<1–7
Near pager antenna	—	—	350	—	—	—	—
1–2 m laterally from pager antennas	—	50–75	—	—	—	—	—
Near horizontal boom for pager antenna	—	—	570	—	—	—	—
Near vertical support pole for pager antenna	—	—	60–90	—	—	—	50–90
1 m from vertical support pole for pager antenna	—	—	—	—	—	—	15–19
Beneath GSM sector antennas	20	—	—	—	—	≤11	—
Behind GSM sector antennas	24	—	—	—	≤8	≤11	≤12
Adjacent to GSM sector antennas	—	—	—	—	—	23	—
In front of GSM sector antennas	30	—	—	—	—	72	—
Behind microwave dish antennas	—	—	—	≤11	—	≤9	—
In front of microwave dish antennas	<6	—	75^a^	—	—	—	—
Near unidentified VHF/UHF antennas	—	—	270	—	—	13	—
Near protective barrier around perimeter of roof	—	—	180	—	—	—	—

^a^The electric field strength measured at this location was likely to have been largely due to other nearby VHF/UHF antennas rather than the 1.2 m microwave dish.

### VHF/UHF broadcast sites

A total of seven sites were visited. The sites each hosted at least one or more of the following transmitters: analogue television, digital television, national FM radio, local FM radio and DAB. Also in place were telecommunication systems including GSM base stations, wide-area paging and point-to-point microwave links.

Where UHF television transmitters were installed, signals were usually transmitted via arrays of antennas enclosed within weatherproof cylinders at the top of the structure. Measurements taken within the vicinity UHF antennas are summarised in Table 3. The VHF radio signals were transmitted via arrays of dipole antennas situated at the top of the tower and were generally arranged in three or four columns. The spot measurements taken in the vicinity of these antennas are summarised in Table 4.

**Table 3.** Electric field strength measured in the vicinity of UHF television antennas.

General location	Details	Electric field strength (V m^–1^)		
		Site 2	Site 4	Site 5
In between two antenna arrays	Ambient level	—	—	60–100
Top platform, just beneath main analogue array (four channels)	Ambient level	40–90	10–20	—
	Close to steelwork	—	25	—
	Close to splitter	>270	—	—
Inside secondary analogue array (single channel)	Ambient level	—	40	—
	Close to ladder	—	60	—
	Close to feeders	—	90	—
Inside digital array	Ambient level^a^	—	20–30	—

^a^No localised field strengths materially exceeding the ambient level were found in this region.

**Table 4.** Electric field strength measured in the vicinity of VHF broadcast radio antennas.

General location	Details	Electric field strength (V m^–1^)					
		Site 1	Site 2	Site 3	Site 4	Site 6	Site 7
Platform above main array (four channels)	Ambient level	50–	100	—	—	—	—
	Near splitter	100–	190	—	—	—	—
Platform beneath main array	Ambient level	30	60	—	20	—	—
	Near ladder	—	—	—	40–80	—	—
	Near feeders	—	—	—	200	—	—
	Near steelwork	—	—	—	300	—	—
Inside main array	Ambient level	120	150–250	—	—	—	20–
	Near ladder	270	—	—	—	—	25
	Near steelwork	390	—	—	—	—	—
	Close behind antennas	240	480	—	—	—	30
	Near splitter	450	—	—	—	—	—
	0.5 m outside structure	—	—	—	—	—	—
							40–50
Adjacent to local FM radio antennas (single channel)	Ambient level	—	—	20–30	15–25	—	—
	Edge of structure	—	—	50	—	—	—
	1 m outside structure	—	—	95	—	—	—
	Near feeders	—	—	—	150	—	—
Platform between local FM radio antennas	Ambient level	—	—	—	—	30–40	—
	Near steelwork	—	—	—	—	100	—
	Near splitters	—	—	—	—	200	—
Platform on outside of structure beneath local FM radio antennas	Ambient level	—	—	—	—	10–50	—
	Near steelwork	—	—	—	—	80	—

### Summary of exposure by site

Staff from seven companies participated: four mobile phone operators and three broadcasters, and a total of 124 completed exposure records were obtained. Here, measures of central tendency are presented as produced by the software program UnCensor 4.0^19^.

**Figure 1.**
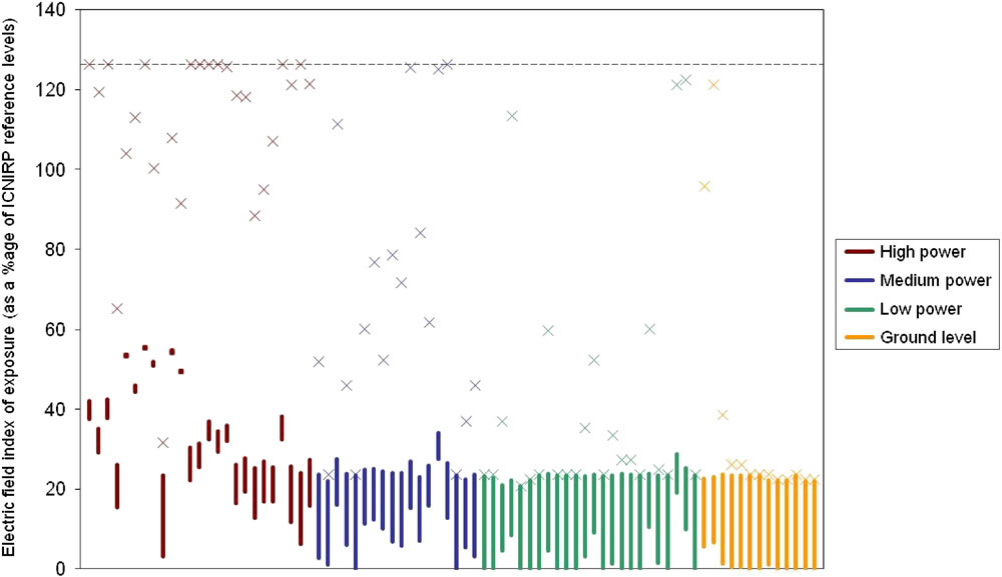
Root mean square and maximum electric field exposure indices at various categories of site. The maximum exposures are presented as a cross. The broken line indicates the upper limit of detection of the ESM-20.

The novel treatment of the data produced a rms analysis. The rms and maximum electric field strength were calculated across four types of site defined by the most powerful antenna at that site. High-power sites are UHF or VHF broadcast sites where powers were in excess of 1 kW. Medium power sites are those with UHF or VHF transmitting powers in the range of 10–1000 W or sites with paging antennas. Low-power sites are those where the most powerful transmitters were associated with GSM base stations where transmitting powers are <10 W per channel. Ground-level sites are where employees worked only at ground level. These data are summarised in Figure 1 where rms exposures are denoted by vertical lines. RF levels above the instruments upper threshold were assigned the upper threshold value (i.e. 120% of ICNIRP). The bottom of each line indicates the rms if it is assumed that all measurements below the threshold of detection were zero.

### Work activity questionnaires

Data from a total of 1253 questionnaires completed by 213 individuals were collated. Job titles were placed into one of five categories, defined in conjunction with the participating companies and reflective of their core role. Riggers working for broadcasters spend some four times longer working in exposed areas than their counterparts working for mobile phone operators. Those working in Antenna Support spend similar time working in exposed areas across both sectors of the industry. The data for both operators and broadcasters are summarised in Table 5.

**Table 5.** Summary of work activity questionnaires by job category and sector.

	Antenna support	General maintenance worker	Occasional climber	Rigger	Satellite support
*Operators*					
Number of individuals	46	3	5	5	
Number of questionnaires	222	15	25	44	
Average working hours per shift	8.2	7.5	8.7	8.7	
Average working hours in exposed areas per shift	0.5	0.0	0.1	0.3	
*Broadcasters*					
Number of individuals	10	4	10	61	5
Number of questionnaires	62	171	126	563	25
Average working hours	7.6	7.8	8.0	8.8	9.3
Average working hours in exposed areas	0.6	0.3	0.0	1.2	0.0

### Exposure versus job group

In combining the findings from the work activity questionnaires with the exposure data stratified by type of site, it is clear that riggers working in broadcast companies are more likely to experience higher RF exposure than other groups of workers. In addition, they also spend more time in exposed locations. By comparison workers employed by operators attending singly located antennas experience far lower levels of exposure than their counterparts in the broadcast industry, levels that are typically below the detection level of the monitors used in this study.

## DISCUSSION

Using data aggregated from personal monitor measurements of employees within broadcasters and mobile network operators, an estimate of exposure by site for both groups was produced. Linking personal exposure measurements at each site to work activity diaries from staff at each company shows that riggers in broadcast companies receive the highest exposure and for the longest duration. The least exposed are those workers whose sole exposure is from working at ground level or otherwise visiting singly located mobile phone antennas.

### Strengths and limitations

The support of key staff at each company meant the research team were able to gather comprehensive and representative data on work patterns and personal exposure. This has allowed for the first time to begin the process of providing meaningful estimates of exposure by job group across sector and site.

The sensitivity of the Wandel & Goltermann monitor used for the majority of the study was limited by its internal noise, which can reach as high as 25% of the reference levels and led to an overestimation of exposure at low-powered sites. Personal monitoring technology has improved in its sensitivity over the period since this study was done^20^. The monitor produces a shaped response designed to offer practical assistance to the wearer of their compliance with ICNIRP guidelines and does not allow further interrogation of the data. However some context for the environments has been provided, it was investigated through a series of spot measurements using broadband survey metres.

The numbers of completed work activity questionnaires varied between organisations. Social desirability reporting can introduce bias in completing questionnaires^21^, though there is no reason to believe that employees within one industry would be more prone to this than another and so relative differences would remain.

### Specific findings

Previous measurements in telecom and broadcasting have indicated that routine exposure is generally low^16^ and though some spot measurements around the VHF antennas did exceed 100 V m^−1^ these were made at the rear of antennas close to steelwork where re-radiation can occur or at splitters where leakage was acknowledged. All of these measurements were made in the highly localised conditions of near-field radiation and are not necessarily indicative of whole-body exposure. However, evidence of occupational exposure is scant and there has been only one study that tried to explore differences in exposures between groups of workers in these industries^15^. Our work provides compelling evidence that in the UK those working on broadcast towers are more exposed to higher electric fields despite the strictly controlled working environment. This may be due to the busy nature of many of the taller structures where broadcast antennas are sited and not necessarily a result of the more powerful antennas, which would typically be powered down or turned off prior to the structure being accessed.

While the relationship between occupations involving exposure to RF and a number of health outcomes (especially cancer and reproductive outcomes) has been the subject of a number of epidemiological studies, the lack of accurate exposure data is a major deficiency^22^. Traditionally occupational exposures have been assessed through the measurement of electric and magnetic field strength using portable hazard survey instrumentation^23–25^. However, the worker's exposure depends on the source's power, modulation scheme and field pattern, as well as the worker's behaviour and adherence to proper safety practice^16^.

More recently, personal monitors have been recommended as the best means of assessing individual exposures of those working in the telecommunication and broadcasting industries^26, 27^. Their convenience means that they are suited for use in future epidemiological studies^14, 16^.

There have been several calls for the use of personal monitors in assessing occupational exposure^14, 28^, despite the fact that electric and magnetic fields can be perturbed by the presence of the human body^28–31^. The evidence presented here appears to demonstrate that they are able to provide robust measures of personal exposure. Since this work was conducted a number of more sophisticated monitors^20^ more sensitive and able to record data for a longer period of time have emerged. In addition, statistical analyses^32, 33^ have emerged that can account for missing data points that these were experienced.

Also relevant to the assessment of occupational exposure to RF is the prevalence of the sophisticated ‘smartphone’ technology, which has already been used to successfully measure personal exposure in other areas^34^. The comparatively advanced computing capabilities this technology offers include global positioning services and barometry, allowing accurate measurements of an individual's height above ground^35^. Utilising this functionality means that data on antennas at a given site could be combined with the height of the individual during specific periods of time to annotate personal measurements and produce a more precise exposure metric.

### Future research

In Europe, the need for the identification of source-based measurement data and the importance of historical exposure has been recognised^36^. In the UK, the National Register of RF Workers will use such data to inform exposure categories in future analyses^37^. Meanwhile across the developing world, where the reliance on RF-based industries continues to grow^38^, the number of employees occupationally exposed to RF continues to increase. This work demonstrates that it is possible to produce high-quality exposure estimates based on a combination of data from monitors and questionnaires that can be used to inform future epidemiological analyses.
